# 青蒿琥酯对肺癌A549细胞侵袭能力及ICAM-1、MMP-9表达的影响

**DOI:** 10.3779/j.issn.1009-3419.2013.11.01

**Published:** 2013-11-20

**Authors:** 献珊 陈, 坤元 韩, 锋夏 陈, 从明 吴, 伟炜 黄

**Affiliations:** 1 570311 海口，海南省人民医院胸外科 Department of Thoracic Surgery, Hainan People' s Hospital, Haikou 570311, China; 2 570311 海口，海南省人民医院老年病科 Department of Geriatrics, Hainan People' s Hospital, Haikou 570311, China; 3 570311 海口，海南省人民医院普外科 Department of General Surgery, Hainan People's Hospital, Haikou 570311, China

**Keywords:** 青蒿琥酯, 肺肿瘤, 侵袭, ICAM-1, MMP-9, Artesunate, Lung neoplasms, Invasion, ICAM-1, MMP-9

## Abstract

**背景与目的:**

近年来研究发现抗疟药青蒿琥酯对肺癌细胞具有抑制效应，但其作用机制尚未完全阐明。本研究旨在探讨青蒿琥酯对肺癌A549细胞侵袭能力的影响。

**方法:**

MTT实验观察青蒿琥酯对体外培养的A549细胞增殖抑制作用；Transwell小室侵袭实验检测青蒿琥酯对体外A549细胞侵袭转移能力影响；构建A549细胞裸鼠皮下移植瘤模型，观察青蒿琥酯对A549细胞移植瘤的抑制作用，并采用Western blot检测A549细胞移植瘤ICAM-1和MMP-9蛋白表达的变化。

**结果:**

MTT实验表明1.25 μg/L-5 μg/L的低浓度青蒿琥酯对A549细胞增殖无明显抑制作用。Transwell小室体外侵袭实验表明，与对照组（96.33±6.41）比较，1.25 μg/L青蒿琥酯即可抑制A549细胞侵袭转移（75.43±4.37, *P* < 0.05）。移植瘤实验表明10 mg/kg青蒿琥酯不能抑制裸鼠A549细胞移植瘤的生长（*P* > 0.05），但是可降低ICAM-1和MMP-9蛋白表达量（*P* < 0.05）。

**结论:**

青蒿琥酯可抑制A549细胞侵袭潜能，其机制可能与降低ICAM-1和MMP-9表达有关。

## Abstract (en)

**Background and objective:**

Artesunate, an anti-malarial drug, elicits an inhibitory effect on pulmonary carcinoma. However, the mechanisms of artesunate activity on pulmonary carcinoma have not been completely elucidated. The aim of this study is to investigate the effect of artesunate on the invasion of human lung adenocarcinoma A549 cells.

**Methods:**

The inhibitory effect of artesunate on the proliferation and invasion of A549 cells was determined *in vitro* by MTT assay and transwell chamber invasion assay, respectively. A nude mouse model of human lung A549 cell xenograft tumor was established. The inhibitory effect of artesunate on the tumor of the mouse model as well as ICAM-1 and MMP-9 protein expressions were determined by Western blot.

**Results:**

A low dose of artesunate ranging between 1.25 μg/L and 5 μg/L did not significantly inhibit the proliferation of A549 cells *in vitro*. By contrast, 1.25 μg/L artesunate inhibited the invasion of A549 cells *in vitro* as determined by transwell chamber invasion assay (96.33±6.41 *vs* 75.43±4.37, *P* < 0.05). Although 10 mg/kg artesunate did not significantly inhibit A549 xenograft tumor proliferation (*P* > 0.05), artesunate decreased the ICAM-1 and MMP-9 protein levels in the mouse model (*P* < 0.05).

**Conclusion:**

Artesunate could inhibit the invasion of human lung adenocarcinoma A549 cells by possibly downregulating ICAM-1 and MMP-9 expressions.

肺癌占全球癌症死亡的首位。其中非小细胞肺癌（non-small cell lung cancer, NSCLC）占肺癌总数超过80%，容易发生侵袭和转移，多数患者死于转移和复发。深入了解肺癌侵袭转移机制，并探讨其防治策略尤为必要。近年来研究^[1, 2]^发现青蒿素及其衍生物除了具有抗疟作用外，对多种肿瘤细胞在体外具有杀伤或抑制作用，但其作用机制尚不十分清楚。多项研究^[3, 4]^证实青蒿素衍生物之一—青蒿琥酯可抑制肺癌细胞增殖，并诱导其凋亡。本研究继续观察低浓度青蒿琥酯对肺癌A549细胞侵袭转移能力的影响和对细胞间粘附分子-1（intercellular adhesion molecule-1, ICAM-1）、基质金属蛋白酶-9（matrix metalloproteinase-9, MMP-9）蛋白表达的影响，以期为NSCLC治疗提供新思路。

## 1 材料与方法

### 1.1 细胞、动物及试剂

人NSCLC A549细胞株购于中南大学肿瘤研究所。SPF级BALB/C-nu/nu品系裸鼠购于中科院上海实验动物中心。青蒿琥酯购于桂林南药股份有限公司（批准文号H-10930195），用5%碳酸氢钠注射液溶解超滤除菌后，再用RPMI-1640培养液稀释成不同终浓度备用。RPMI-1640培养基和胎牛血清购于浙江天杭生物科技有限公司。胰蛋白酶、MTT（噻唑蓝）和DMSO（二甲基亚砜）购于美国Sigma公司。Transwell侵袭小室购自美国Costar公司。ICAM-1和MMP-9兔多抗购于美国Santa Cruz公司。

### 1.2 细胞培养

将A549细胞用RPMI-1640培养液培养，内含10%胎牛血清、青霉素100 U/mL、链霉素100 U/mL。置于37 ℃、5%CO_2_、饱和湿度环境下培养。

### 1.3 MTT实验

取对数生长期的A549细胞，调细胞浓度为2×10^5^个/mL，以每孔200 μL接种于96孔板，每组6复孔。分别加入终浓度为0 μg/L、1.25 μg/L、2.5 μg/L、5 μg/L、10 μg/L和20 μg/L的青蒿琥酯50 μL，置37 ℃、5%CO_2_培养箱中培养24 h。离心去上清，每孔加5 mg/mL的MTT 20 μL，37 ℃培养4 h后，加入现配的DMSO，振荡溶解，酶标仪测570 nm处吸光度。计算细胞增殖抑制率。

### 1.4 Transwell小室体外侵袭实验

取对数生长期的A549细胞，分别加0 μg/L、1.25 μg/L、2.5 μg/L和5 μg/L的青蒿琥酯作用24 h，胰蛋白酶消化，调细胞浓度为2×10^5^个/mL，各组分别吸取200 μL接种于上层小室，将其置于灭菌的24孔板。下室加入含10%小牛血清的RPMI-1640培养液。常规培养24 h。将上室面的Matrigel和细胞用湿棉签擦掉，膜用甲醇固定10 min，苏木精染色，高倍镜下计数穿过微孔膜的细胞数。每组细胞随机计数10个视野，求其平均值。实验重复3次。

### 1.5 裸鼠A549细胞移植瘤生长抑制实验

于裸鼠右后肢皮下接种A549细胞0.2 mL（约含活细胞数1×10^6^个），约1周后长出肿块。取已成瘤小鼠20只，随机分成4组。参考文献[5]分别腹腔注射低浓度青蒿琥酯10、20和40 mg/kg，对照组注射等量生理盐水。每日1次，连续注射14天。于最后一次给药治疗12 h后无痛处死小鼠，取出移植瘤并称重。计算抑瘤率。

### 1.6 Western blot实验

分别将各组移植瘤标本匀浆，提取总蛋白，蛋白定量，取15 μL样品加入等体积2×上样缓冲液，煮沸5 min备用。取100 μg蛋白样品进行SDS-PAGE凝胶电泳后，电转至硝酸纤维素膜，脱脂奶粉封闭液孵育2 h，加入相应一抗4 ℃过夜（β-actin为内参），漂洗后与辣根过氧化物酶标记的二抗结合，化学发光显色。IPP光密度分析软件进行目的条带的表达检测及分析。

### 1.7 统计学方法

实验数据以Mean±SD表示，采用SPSS 16.0统计软件，两均数比较经方差齐性检验后，方差齐者用*t*检验。多组间数值资料比较采用方差分析。以*P* < 0.05为差异具有统计学意义。

## 2 结果

### 2.1 青蒿琥酯对A549细胞的增殖抑制作用

青蒿琥酯对A549细胞的生长具有抑制作用。青蒿琥酯终浓度 > 5 μg/L时，对A549细胞生长抑制作用明显，差异有统计学意义（*P* < 0.01）；青蒿琥酯终浓度≤5 μg/L时，在24 h内对A549细胞的生长无明显毒性作用，差异无统计学意义（*P* > 0.05），A549细胞存活率超过90%（表 1）。后续Transwell小室体外侵袭实验采用较低浓度青蒿琥酯（≤5 μg/L）。

**1:** 青蒿琥酯对A549细胞增殖率的影响（Mean±SD, *n*=6） The proliferation-inhibiting effect of artesunate on A549 cells (Mean±SD, *n*=6)

Concentration (*μ*g/L)	OD (A_570_ nm)	Inhibition rate (%)
0	0.795±0.142	-
1.25	0.768±0.123	96.6
2.5	0.745±0.112	94.1
5	0.694±0.153	90.6
10	0.589±0.096^*^	74.1
20	0.402±0.028^*^	50.6
^*^P < 0.01, when compared with 0 *μ*g/L artesunate group.		

### 2.2 青蒿琥酯抑制A549细胞侵袭转移

Transwell体外侵袭实验结果显示，与对照组穿过小室的细胞数（96.33±6.41）相比，青蒿琥酯1.25 μg/L组、2.5 μg/L组及5 μg/L组的侵袭转移细胞数明显减少，分别为（75.43±4.37）（*P* < 0.05）、（61.43±3.61）（*P* < 0.01）和（39.31±3.41）（*P* < 0.01）（图 1）。

**1:**
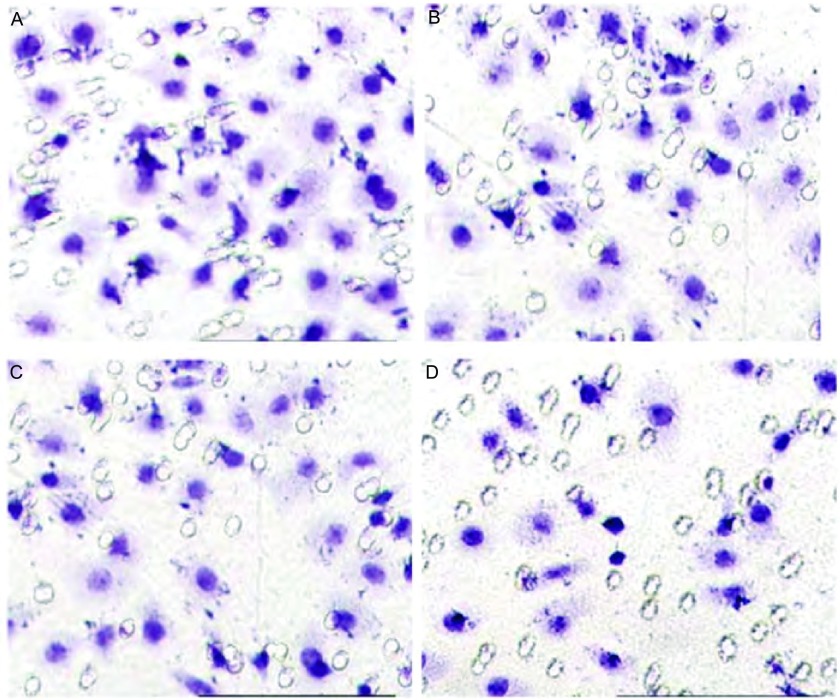
不同浓度青蒿琥酯对肺癌A548细胞侵袭、迁移的影响。A：0 *μ*g/L青蒿琥酯组；B：1.25 *μ*g/L青蒿琥酯组；C：2.5 *μ*g/L青蒿琥酯组；D：5 *μ*g/L青蒿琥酯组。 Effects of artesunate with different doses on migration and invasion of lung cancer A459 cells. A: 0 *μ*g/L artesunate group; B: 1.25 *μ*g/L artesunate group; C: 2.5 *μ*g/L artesunate group; D: 5 *μ*g/L artesunate group.

### 2.3 青蒿琥酯对裸鼠A549细胞移植瘤的影响

经低浓度青蒿琥酯处理后，各剂量组移植瘤瘤重均较对照组减轻。其中青蒿琥酯20 mg/kg组（0.52±0.05）g和40 mg/kg组（0.37±0.03）g的瘤重与对照组（0.69±0.05）g相比有统计学差异（*P* < 0.01）；10 mg/kg青蒿琥酯组瘤重（0.62±0.04）g与对照组相比无统计学差异（*P* > 0.05）（图 2）。随青蒿琥酯剂量的增加，对移植瘤的抑制率有增大趋势。各剂量组的抑瘤率分别为8.69%（10 mg/kg组）、24.74%（20 mg/kg组）和43.09%（40 mg/kg组）。

**2:**
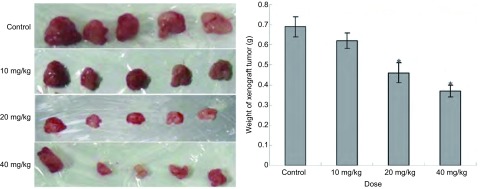
不同剂量青蒿琥酯对A549细胞移植瘤瘤重影响（Mean±SD, *n*=5）（^*^P < 0.01, 与对照组比较） The effect of artesunate with different doses on the heavy of A549 cell xenograft tumor (Mean±SD, *n*=5) (^*^P < 0.01, when compared with control group)

### 2.4 青蒿琥酯对裸鼠A549细胞移植瘤ICAM-1和MMP-9蛋白表达影响

Western印迹结果显示，经青蒿琥酯处理后，10 mg/kg青蒿琥酯组即开始出现ICAM-1和MMP-9蛋白表达量降低。与对照组相比，ICAM-1蛋白表达分别降低16.67%（10 mg/kg组）、40.48%（20 mg/kg组）和58.91%（40 mg/kg组）；MMP-9蛋白表达分别降低21.05%（10 mg/kg组）、41.52%（20 mg/kg组）和66.31%（40 mg/kg组），差异有统计学意义（*P* < 0.05）（图 3）。

**3:**
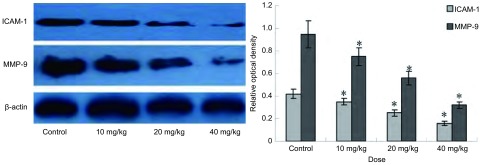
不同剂量青蒿琥酯对A549细胞移植瘤ICAM-1和MMP-9蛋白表达影响（Mean±SD, *n*=6）（^*^P < 0.05, 与对照组比较） The effect of artesunate with different doses on the ICAM-1 and MMP-9 expression in A549 cell xenograft tumor (Mean±SD, *n*=6) (^*^P < 0.05, when compared with control group)

## 3 讨论

青蒿素是我国科学家开发的具有完全知识产权的抗疟药。青蒿琥酯是青蒿素的衍生物之一，具有更好的水溶性和抗疟效果。研究^[2, 6]^表明青蒿素及其衍生物除有抗疟作用外，还具有抗肿瘤、抗炎、抗纤维化等生物学作用。目前青蒿素及其衍生物表现出的强大抗肿瘤活性越来越引起国内外学者注意。美国癌症协会的研究^[1]^表明：青蒿琥酯对结肠癌细胞、白血病细胞、黑色素瘤细胞、乳腺癌细胞等55种恶性肿瘤细胞具有细胞毒性作用。多项研究^[3, 4]^亦证实青蒿琥酯可以剂量依赖方式抑制体外培养的肺癌细胞增殖，并诱导其凋亡。本研究以较低浓度青蒿琥酯作用于裸鼠A549细胞移植瘤模型，观察青蒿琥酯对A549细胞的体内抗瘤效应。结果发现较低浓度的青蒿琥酯即对移植瘤有一定抑制作用。进一步提示青蒿琥酯可能是一种潜在的抗肺癌药物选择。

肿瘤的侵袭和转移是导致肿瘤患者死亡的主要原因。NSCLC转移复发率更是居于原发性恶性肿瘤的前列。从分子水平深入了解肺癌侵袭转移机制，并探讨其防治策略尤为必要。肿瘤的侵袭转移是一个多阶段的复杂过程，从分子水平可概括为粘附、蛋白降解和移动三个步骤。肿瘤细胞首先粘着于细胞外基质、血管内皮、靶器官细胞等处。然后分泌水解酶，降解和破坏细胞外基质及毛细血管基底膜等。突破限制后浸入周围组织器官或进入血循环或淋巴系统，最终形成转移灶。目前关于青蒿琥酯对恶性肿瘤细胞侵袭转移能力影响较少有报道。在炎症、纤维化等的研究^[7-9]^中发现，青蒿琥酯具有调节蛋白水解酶表达，影响细胞外基质合成及降解的作用。鉴于此，本研究首先通过体外实验，得到青蒿琥酯对A549细胞增殖抑制作用的浓度范围，然后以较低浓度青蒿琥酯进行Transwell小室侵袭实验，观察较低浓度青蒿琥酯对体外培养的肺癌A549细胞侵袭能力的影响。结果发现A549细胞侵袭能力随着青蒿琥酯浓度增加而逐渐降低。提示青蒿琥酯对肺癌细胞的抑制作用可能也与其降低肺癌细胞侵袭能力有关。

ICAM-1和MMP-9是肿瘤细胞侵袭转移过程中非常重要的两个细胞因子。ICAM-1表达于肿瘤细胞膜表面，介导肿瘤细胞-内皮细胞、肿瘤细胞-细胞外基质的粘附；同时也介导肿瘤细胞-淋巴细胞粘附，使肿瘤细胞易于随淋巴细胞迁移进入血循环或淋巴循环，增加肿瘤迁移浸润的机会。ICAM-1在多种类型的肿瘤组织中存在异常表达^[10]^。针对NSCLC患者的研究^[11, 12]^发现，ICAM-1在NSCLC组织中高表达，且其表达量与肺癌不同分期、侵袭转移和病理类型等密切相关。本研究亦证实在体内A549细胞移植瘤中存在ICAM-1高表达。提示ICAM-1与肺癌的侵袭转移密切相关。MMPs是降解细胞外基质最重要的酶类，同时还具有促进肿瘤血管生成的作用。MMP-9是MMPs中分子量最大的酶，可降解细胞外基质及基底膜的主要结构蛋白Ⅳ型胶原，在胶质瘤、胃癌、结肠癌等多种侵袭性强的恶性肿瘤中高表达。临床研究发现，MMP-9不光高度表达于肺癌组织中，在患者血清水平亦存在显著升高，是NSCLC患者早期诊断和预后判断的重要参考指标^[13, 14]^。抑制体外培养的H1299肺癌细胞MMP-9表达，则能抑制其早期转移^[15]^。本研究亦发现在体内A549细胞移植瘤中存在MMP-9高表达。鉴于ICAM-1和MMP-9与肺癌侵袭转移的密切关系，本研究观察了较低浓度青蒿琥酯对A549细胞裸鼠皮下移植瘤内ICAM-1和MMP-9表达的影响。结果发现较低浓度青蒿琥酯（10 mg/kg）即可下调移植瘤内ICAM-1和MMP-9表达量，提示青蒿琥酯对肺癌细胞侵袭能力的影响可能与其抑制ICAM-1和MMP-9表达有关。
